# Kinetic isotope effects for fast deuterium and proton exchange rates

**DOI:** 10.1039/c5cp07459b

**Published:** 2016-03-24

**Authors:** Estel Canet, Daniele Mammoli, Pavel Kadeřávek, Philippe Pelupessy, Geoffrey Bodenhausen

**Affiliations:** a Ecole Polytechinque Fédérale de Lausanne , Laboratoire de Résonance Magnétique Biomoléculaire , Batochime , CH-1015 Lausanne , Switzerland . Email: estel.canet@epfl.ch; b Ecole Normale Supérieure – PSL Research University , Département de Chimie , 24 rue Lhomond , 75231 Paris Cedex 05 , France; c Sorbonne Universités , UPMC Univ Paris 06, LBM , 4 place Jussieu , 75005 Paris , France; d CNRS , UMR 7203 LBM , 75005 Paris , France

## Abstract

By monitoring the effect of deuterium decoupling on the decay of transverse ^15^N magnetization in D–^15^N spin pairs during multiple-refocusing echo sequences, we have determined fast D–D exchange rates *k*_D_ and compared them with fast H–H exchange rates *k*_H_ in tryptophan to determine the kinetic isotope effect as a function of pH and temperature.

## Abstract 2

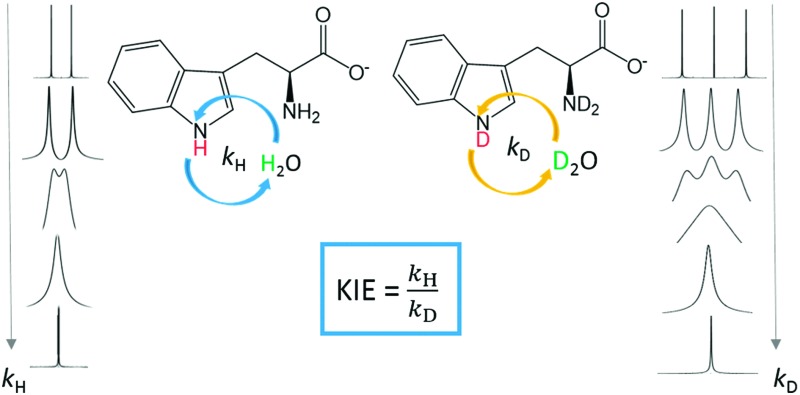
By monitoring the effect of deuterium decoupling on the decay of transverse ^15^N magnetization in D–^15^N spin pairs during multiple-refocusing echo sequences, we have determined fast D–D exchange rates *k*_D_ and compared them with fast H–H exchange rates *k*_H_ in tryptophan to determine the kinetic isotope effect as a function of pH and temperature.

## Introduction

In the parlance of magnetic resonance, chemical exchange is a process where a nucleus undergoes a change of its environment.^1^ The determination of the exchange rates of labile protons can provide valuable insight into both structural and dynamic aspects of a wide range of molecules,^2–4^ such as the opening of base-pairs in nucleic acids and protection factors in protein–ligand complexes.^5,6^ In this paper, we shall focus on measurements of D–D exchange rates *k*_D_ and their comparison with H–H exchange rates *k*_H_ in tryptophan.^7^ The knowledge of kinetic isotope effects, *i.e.*, of the ratio *k*_H_/*k*_D_ that expresses the reduction of D–D exchange rates *k*_D_ compared to H–H exchange rates *k*_H_, may contribute to the characterization of reaction mechanisms.^8,9^ The kinetic isotope effect can give insight into the stability of hydrogen-bonded secondary structures in biomolecules.^10^ In this work, we shall consider exchange processes involving labile D^N^ deuterons and H^N^ protons that are covalently bound to the nitrogen atom in the indole ring of tryptophan.†
†Despite IUPAC recommendations, we use the notation ^2^D rather than ^2^H.


## Experimental section

**Fig. 1:**
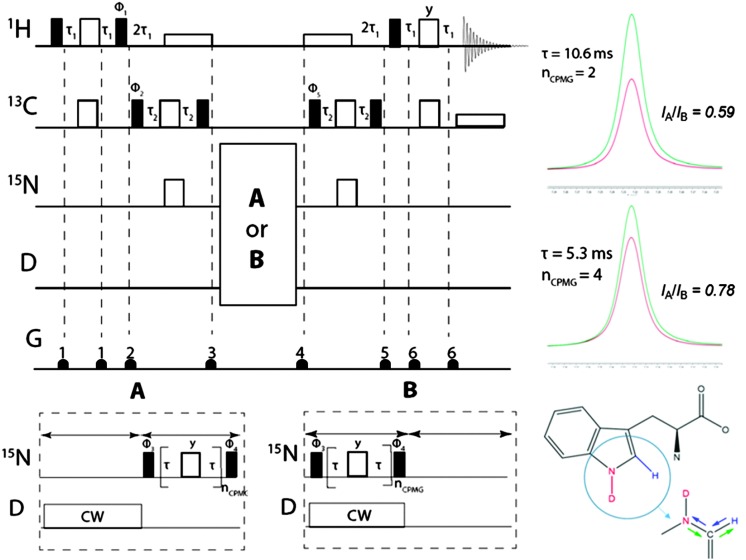
(left) Pulse sequence for measurements of the indole D–D exchange rate *k*_D_. The π/2 and π pulses are represented by narrow filled and wide open rectangles respectively while wide open rectangles depict decoupling sequences. All phases are along the *x*-axis unless indicated otherwise. The phase cycling is: *Φ*_1_ = 16(*y*), 16(–*y*); *Φ*_2_ = *x*, –*x*; *Φ*_3_ = 2(*x*), 2(–*x*); *Φ*_4_ = 4(*x*), 4(–*x*); *Φ*_5_ = 8(*x*), 8(–*x*) and the receiver phase is *Φ*_rec_ = *x*, –*x*, –*x*, *x*, 2(–*x*, *x*, *x*, –*x*), *x*, –*x*, –*x*, *x*, –*x*, *x*, *x*, –*x*, 2(*x*, –*x*, –*x*, *x*), –*x*, *x*, *x*, –*x*. The delays are: *τ*_1_ = 1/(4J_CH_) = 1.56 ms and *τ*_2_ = 1/(4J_CN_) = 16 ms. The gradient pulses G that bracket π-pulses at positions 1 and 6 are of equal strength and polarity to cancel effects of pulse imperfections. The gradients applied at positions 2, 3, 4 and 5 are used to purge any undesired transverse magnetization, as the magnetization of interest is aligned with *z* during the corresponding intervals. (top right) Proton signals of tryptophan at pD 8.7 and *T* = 300 K. The red lines correspond to experiment A without decoupling while the green lines stem from experiment B with deuterium decoupling. (top) *I*_A_/*I*_B_ = 0.59 with *τ* = 10.6 ms and *n*_CPMG_ = 2. (bottom) *I*_A_/*I*_B_ = 0.78 with *τ* = 5.3 ms and *n*_CPMG_ = 4. (bottom right) Consecutive coherence transfer steps from the blue ^1^H to the red ^15^N *via* ^13^C and back in ^2^D, ^13^C, and ^15^N labelled tryptophan.

We have adapted to the case of deuterium (spin *S* = 1) a scheme that was originally designed to determine fast exchange rates of protons^7,11,12^ (spin *S* = 1/2) by monitoring the effect of deuterium decoupling on the decay of transverse ^15^N magnetization during multiple-refocusing sequences (CPMG).‡
‡We use the symbol ^2^D when referring to isotopes as in the expressions ^1^ *J*(^1^H,^15^N) or ^1^ *J*(^2^D,^15^N).
^13,14^ The modified pulse sequence is shown in Fig. 1. The scheme requires isotopic enrichment with ^15^N and ^13^C, since the ^15^N coherence is excited by transfer from neighboring protons through two successive INEPT transfer steps *via* ^1^ *J*(^13^C,^1^H) and ^1^ *J*(^15^N,^13^C). The decay of the ^15^N coherence is monitored indirectly after transferring the coherence back to the proton of origin. The ^15^N,^13^C-labelled isomers of tryptophan are dissolved in either D_2_O or H_2_O to determine the kinetic isotope effect *k*_H_/*k*_D_ of the following reactions:

1 N–D + D′^+^ → N–D′ + D^+^ rate *k*_D_

2 N–H + H′^+^ → N–H′ + H^+^ rate *k*_H_

where *k*_D_ and *k*_H_ are the pseudo-first order rate constants since the concentration of the solvent D_2_O or H_2_O, which is the source of the incoming D′^+^ or H′^+^ ions, is constant and much higher than the concentration of the solute.§
§We shall refer to H or D for atoms that appear in molecular formulae and to H^N^ or D^N^ in N–H and N–D groups. For the Cartesian components of angular momentum operators, we have used H_*x*_, H_*y*_, H_*z*_, D_*x*_, D_*y*_, D_*z*_, N_*x*_, N_*y*_, N_*z*_, C_*x*_, C_*y*_, C_*z*_ rather than the common notation I_*x*_, R_*x*_, S_*x*_, *etc.*



The first and last parts of the pulse sequence in Fig. 1 lead to a transfer of the magnetization from the blue non-exchanging ‘spy’ proton to ^15^N and back, *via* the adjacent ^13^C nuclei, by two successive pulse sequences for Insensitive Nuclei Enhanced by Polarization Transfer (INEPT).^15^ The first INEPT sequence transforms longitudinal proton magnetization H_*z*_ into two-spin order 2H_*z*_C_*z*_. The second INEPT sequence converts 2H_*z*_C_*z*_ into 2C_*z*_N_*z*_. WALTZ-16 proton decoupling^16^ is used to suppress the evolution under ^1^ *J*(^1^H, ^13^C) during the intervals of the INEPT sequences where the coherence is transferred from ^13^C to ^15^N. The antiphase coherence 2N_*y*_C_*z*_ excited at the beginning of the multiple-refocusing CPMG interval decays in the course of this pulse train. At this point, two variants (A and B) of the experiments must be performed. In experiment B, continuous wave (CW) deuterium decoupling is applied during the CPMG pulse train, while in experiment A the deuterium irradiation is applied for the same duration but prior to the CPMG pulse train in order to avoid differences in temperature.

The remaining coherence 2N_*y*_C_*z*_ is transferred back to the ‘spy’ proton for detection. The intensity of the resulting peak near 7.22 ppm in the proton spectra is proportional to the magnitude of the nitrogen 2N_*y*_C_*z*_ coherence that remains at the end of the CPMG interval. In order to extract *k*_D_ one can determine the ratio *I*_A_/*I*_B_ of the peak intensities recorded without decoupling during the CPMG pulse train (experiment A) and with deuterium decoupling (experiment B). The delay *τ* is defined as one-half of the interval between consecutive nitrogen π-pulses. The *τ* delays need to be long enough to ensure that the ratio *I*_A_/*I*_B_ is significantly different from 1. Typically, values of *τ* = 10.6 or 21.2 ms have been used. The scalar coupling is ^1^ *J*(^15^N,^2^D) = 15.4 Hz, smaller than ^1^ *J*(^15^N,^1^H) = 98.6 Hz by the factor *γ*(^2^D)/*γ*(^1^H) ≈ 0.15, but ^1^ *J*(^15^N,^2^D) is still large enough to act as an efficient vehicle of scalar relaxation.

We can construct the matrix representations of the 4 × 9 = 36 Cartesian operators that span a complete basis set for a system comprising a ^15^N nucleus with spin *I* = 1/2 and a ^2^D nucleus with spin *S* = 1.^17^ When a CPMG multiple echo sequence is applied to the ^15^N spins with an on-resonance *rf* field at the chemical shift of ^15^N, while deuterium decoupling is applied with an amplitude *ω*D1 at an offset *Ω* ^D^ with respect to the chemical shift of ^2^D. The *rf* pulses applied to the ^15^N spins are considered to be ideal. Starting from an operator N_*y*_, coherent evolution leads to the following terms:^18^
3
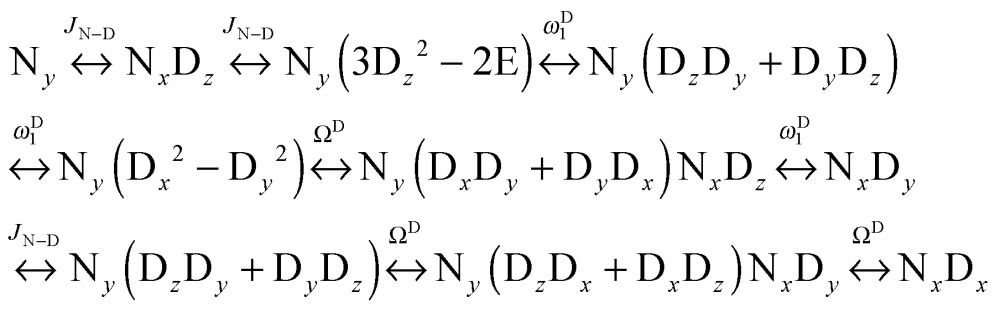
 Therefore the dimension of the basis set can be reduced from 36, leaving only 9 terms:4
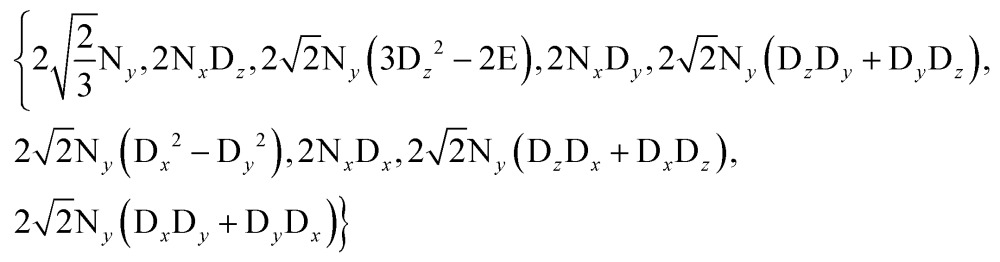
 Note that in the experiments of Fig. 1, the single-quantum coherence at the beginning of the CPMG period is an antiphase operator 2N_*y*_C_*z*_. Since the presence of the C_*z*_ term affects the signal intensities in experiments A and B equally, this C_*z*_ term can be omitted without loss of generality. The solution of the Liouville-von Neumann equation^19^ up to the *n* ^th^ echo is:

5 *σ*(*t* = 2*nτ*) = [exp(–*Lτ*)·*R*_N_·exp(–*Lτ*)]^*n*^*σ*(0)

**Fig. 2:**
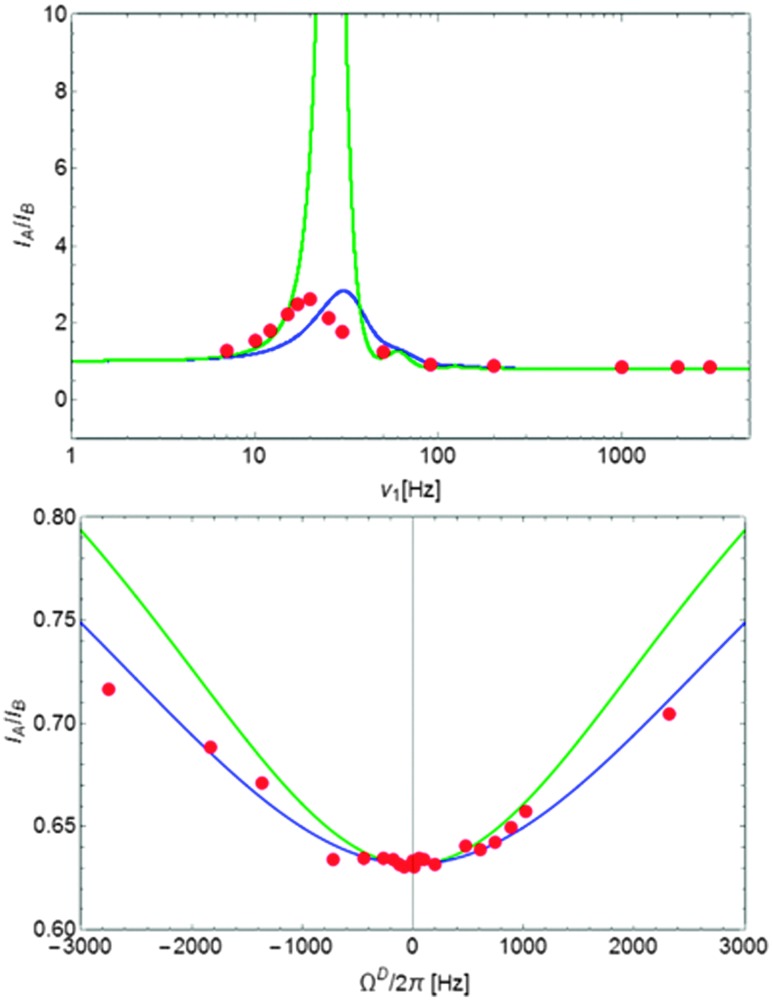
(top) Experimental ratio *I*_A_/*I*_B_ as a function of the amplitude *ν*D1 of the *rf* field applied to the deuterium spins recorded at pD 7.7 and *T* = 300 K with *τ* = 10.6 ms and *n*_CPMG_ = 2. Anomalous ratios *I*_A_ */I*_B_ > 1 only occur when the *rf* amplitude is too low, in particular in the vicinity of ^1^ *J*(^15^N,^2^H). (bottom) Experimental ratio *I*_A_ */I*_B_ as a function of the offset *Ω* ^D^ of the carrier frequency with respect to the deuterium resonance for pD 9.4, *T* = 300 K, *τ* = 10.6 ms, and *n*_CPMG_ = 2. The lines correspond to eqn (9) (top) and eqn (6) (bottom). For the blue lines, we have assumed that different operator products involving deuterium terms have distinct quadrupolar relaxation rates that depend on the spectral density. For the green lines, we have assumed that all deuterium terms have the same relaxation rate. For strong on-resonance *rf* fields, as we have used for the determination of exchange rates, the ratios *I*_A_ */I*_B_ do not change significantly if one assumes a single or several distinct relaxation rates.

The matrix representation of the Liouvillian *L* in the basis of eqn (4) is:6
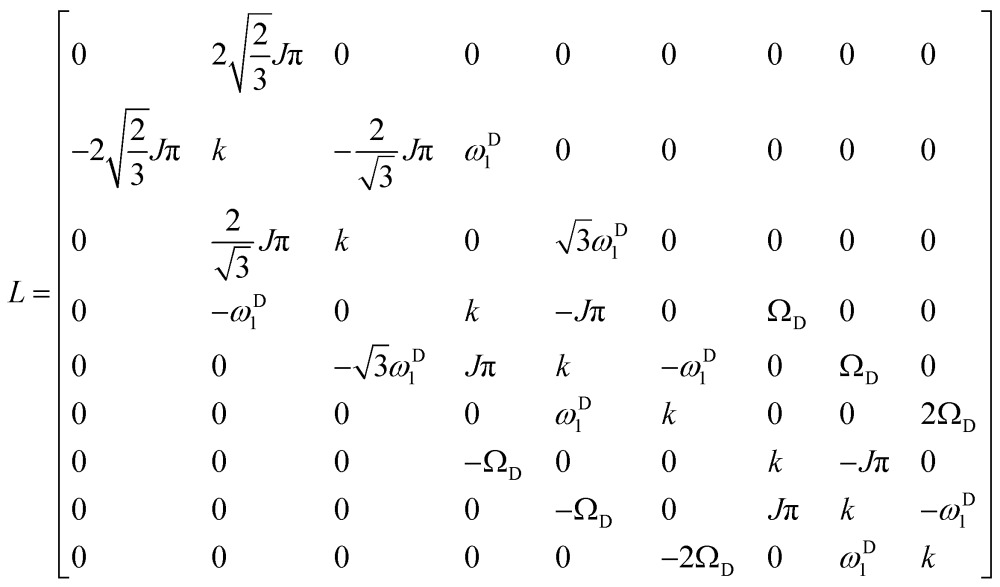
 The matrix representation of *R*_N_ represents a π_*y*_ pulse applied to the ^15^N spins:7
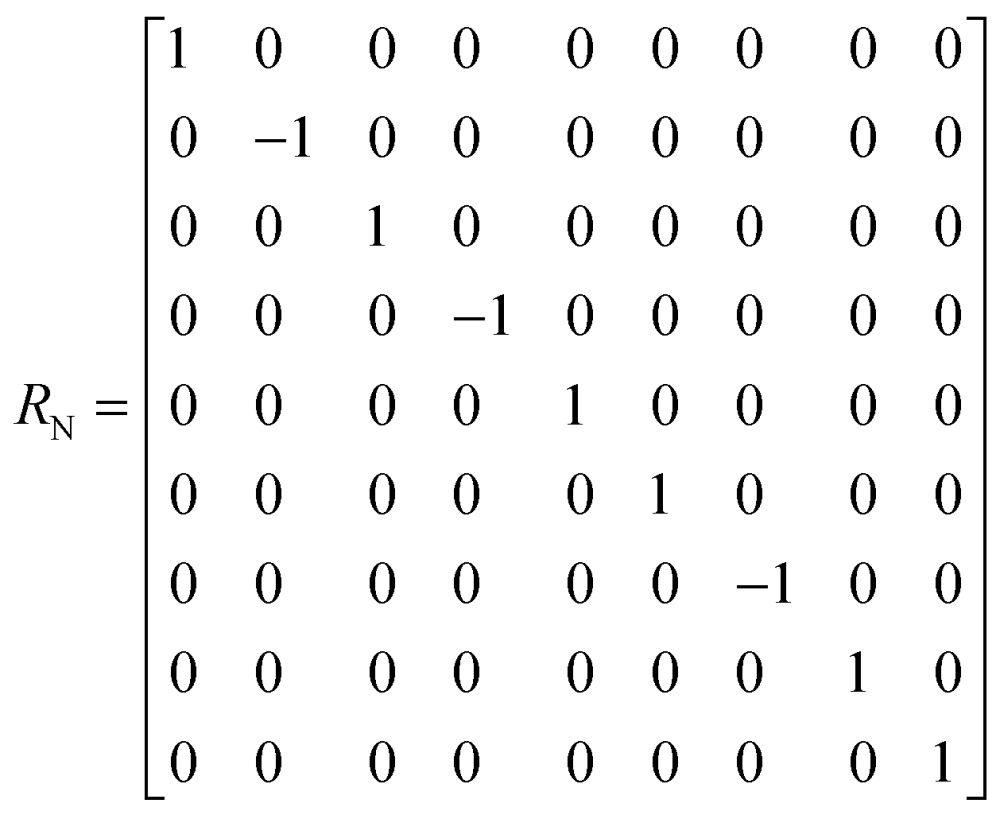
 If the *rf* field for deuterium decoupling is applied on resonance, the evolution of the density operator can be described in a simplified base comprising only 6 product operators:8
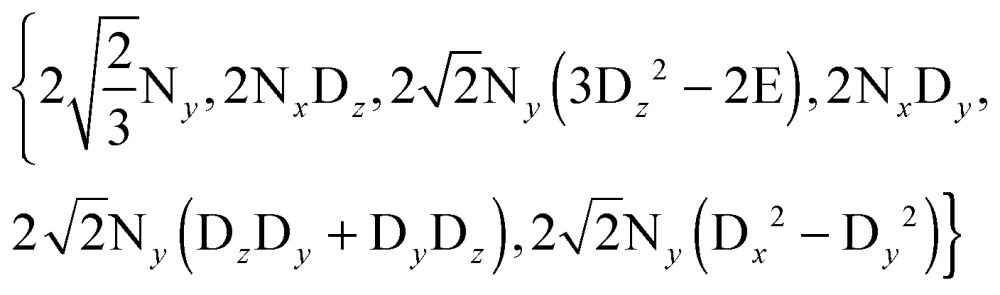
 In this reduced base, the matrix representation of the Liouvillian is:9
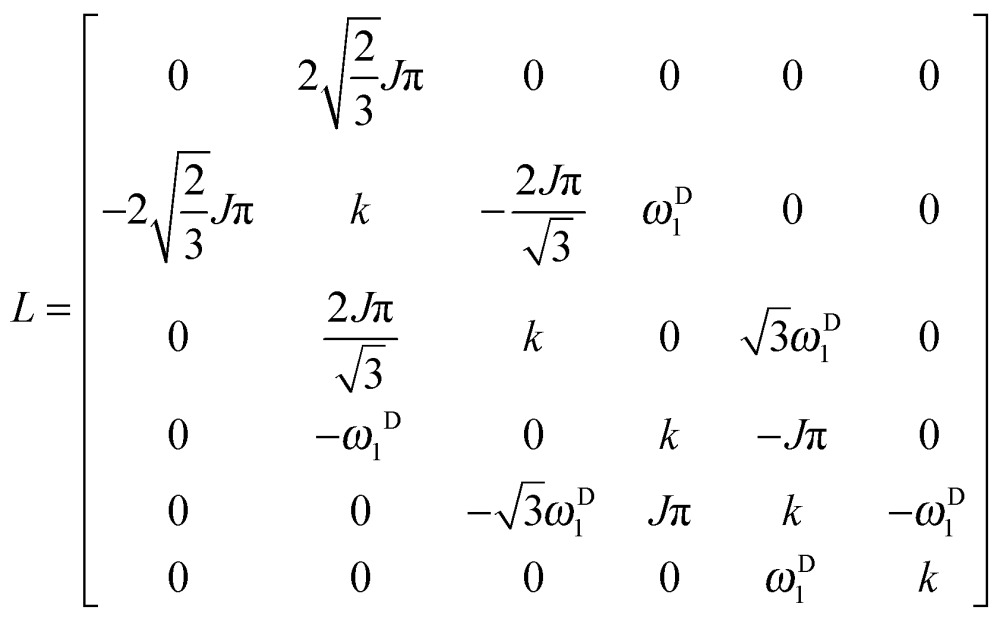
 For the π_*y*_ pulse applied to the ^15^N spins one obtains in this reduced base:10
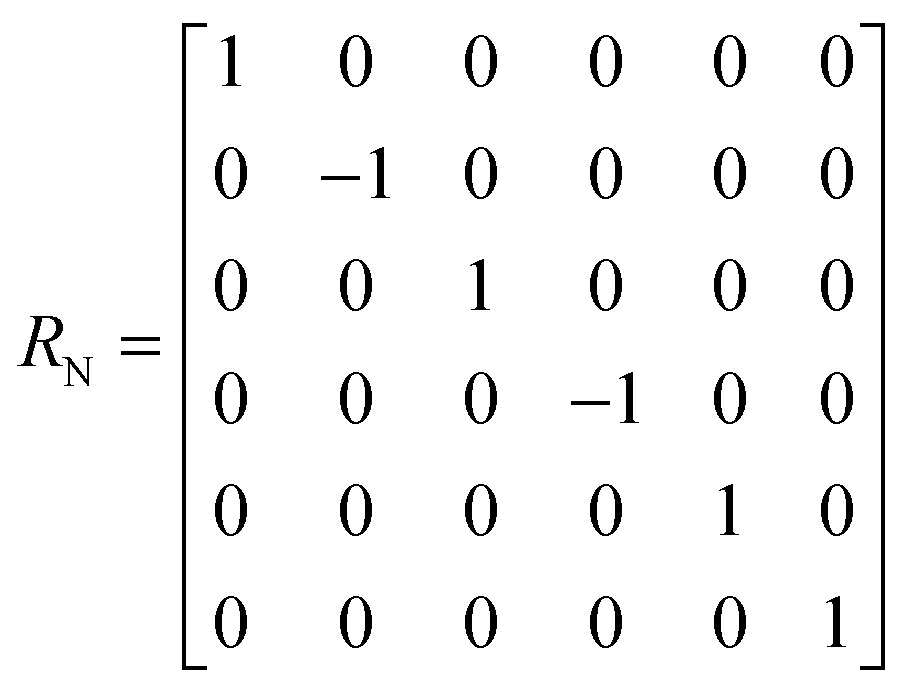
 In the experiment of Fig. 1, the amplitude *ν*D1 of the continuous-wave *rf* field applied to the deuterium spins should be chosen carefully. The higher the *rf* amplitude *ν*D1, the more efficient the decoupling, although one should avoid excessive heating. On the other hand, if the *rf* amplitude is too low, the ratio *I*_A_/*I*_B_ is affected in a manner that can lead to erroneous measurements of the exchange rates. By way of illustration, at pD 7.7 and *T* = 300 K, where the exchange rate is very low (see Table 1), the ratio *I*_A_/*I*_B_ has been determined as a function of the *rf* amplitude for *τ* = 10.6 ms and *n*_CPMG_ = 2. For these experimental conditions, the amplitude can be attenuated as low as *ν*D1 = *ω*D1/(2π) = 100 Hz without affecting significantly the ratio *I*_A_/*I*_B_. For lower amplitudes the ratio is very sensitive to the exact amplitude. An *rf* field with an amplitude *ν*D1 = 3 kHz seems to be a safe value regardless of the exchange rates and can be used for all experiments. The ratio *I*_A_/*I*_B_ also depends on the offset *Ω* ^D^ of the *rf* carrier with respect to the exchanging ^2^D spins, since decoupling becomes less efficient when the carrier is off-resonance. The ratio *I*_A_/*I*_B_ has the smallest value when the carrier coincides with the chemical shift of the exchanging ^2^D spins, *i.e.*, when *Ω* ^D^ = 0 (Fig. 2). The heteronuclear scalar coupling constant ^1^ *J*(^15^N,^2^H) = 15.4 Hz at pD 7.7 was determined experimentally from the doublet in the ^2^H spectrum and corresponds to the expected value ^1^ *J*(^15^N,^2^H) = ^1^ *J*(^15^N,^1^H) *γ*(^2^H)/*γ*(^1^H) with ^1^ *J*(^15^N,^1^H) = 98.6 Hz.

**Table 1:** Pseudo first-order exchange rate constants *k*_D_ [s^–1^] without corrections for contributions due to quadrupolar relaxation as a function of temperature and pD

pD	290 K	pD	300 K	pD	310 K	pD	320 K
1.05	273 ± 21	1.05	491 ±63	1.05	697 ± 110	1.05	1670 ± 250
1.49	56.9 ± 11	1.49	65.9± 13	1.49	81.6 ± 17	1.49	91.6 ± 19
2.18	49.7 ± 4.8	2.18	47.9 ± 4.2	2.18	57.6 ± 11	2.18	61.8 ± 8.7
3.29	39.1 ± 1.9	3.29	30.2 ± 1.8	3.29	26.3 ± 1.5	3.29	23.6 ± 0.7
4.78	36.2 ± 3.0	4.78	26.2 ± 3.3	4.78	20.9 ± 2.9	4.78	16.5 ± 3.2
5.98	37.1 ± 3.1	5.98	27.1 ± 1.9	5.98	22.9 ± 1.9	5.98	18.7 ± 1.4
6.43	38.7 ± 5.7	6.43	28.6 ± 4.2	6.43	23.7 ± 3.1	6.43	19.4 ± 2.6
7.97	41.4 ± 6.4	7.69	31.6 ± 4.3	7.41	27.2 ± 3.4	7.13	23.8 ± 3.1
9.11	82.4 ± 15	8.83	88.1 ± 8.2	8.55	110 ± 11	8.27	182 ± 31
9.68	121 ± 13	9.40	328 ± 17	9.12	507 ± 24	8.84	860 ± 49
10.8	831 ± 42	10.52	1546 ± 90	10.24	2670 ± 330	9.96	4330 ± 380
12.1	3220 ± 840	11.82	8060 ± 2200	11.54	11 800 ± 2600	11.26	18 800 ± 1700
12.97	14 000 ± 3400	12.69	17 600 ± 6300	12.41	40 400 ± 11 000	—	—

All experiments were performed at 14.1 T (600 MHz for ^1^H, 151 MHz for ^13^C, 92 MHz for ^2^H, and –61 MHz for ^15^N) using a Bruker Avance III spectrometer equipped with a cryogenically cooled TXI probe. The samples were prepared by dissolving 20 mM tryptophan (fully ^13^C and ^15^N enriched) in 100% D_2_O buffered with 20 mM citrate, acetate, Tris or phosphate buffer depending on the pH range. We determined *k*_H_ in our earlier work^7^ using 97% H_2_O and 3% D_2_O. The pH was adjusted by DCl or NaOD; the indicated pH values include corrections to take into account that the pH was measured in D_2_O with an electrode calibrated for H_2_O according to the following equation^20^

11 pD = pH_apparent_ + 0.4

## Results and discussion

**Fig. 3:**
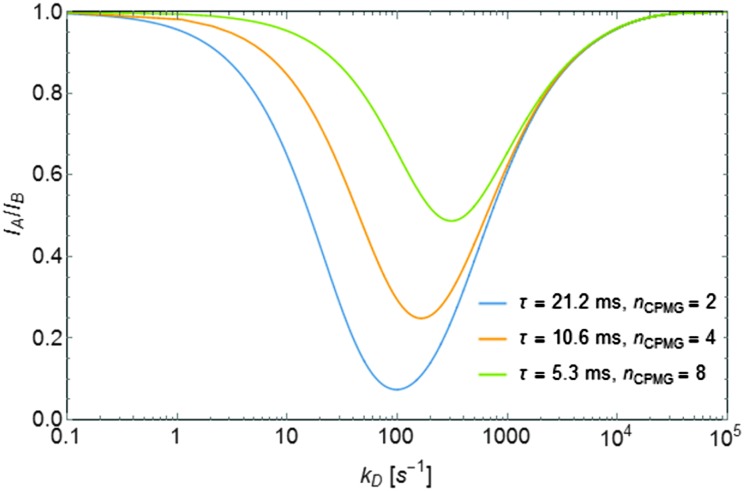
Simulated ratios *I*_A_/*I*_B_ as a function of the exchange rates *k*_D_. The curves correspond to *τ* = 21.2 ms and *n*_CPMG_ = 2, *τ* = 10.6 ms and *n*_CPMG_ = 4, and finally *τ* = 5.3 ms and *n*_CPMG_ = 8, keeping the total time 2*τn*_CPMG_ constant.

**Fig. 4:**
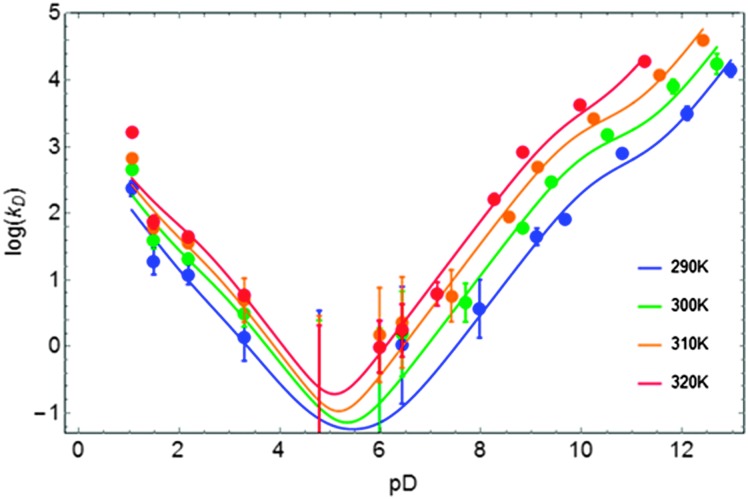
Exchange rate constants *k*_D_ with corrections of Table 2 for the contributions due to quadrupolar relaxation as a function of pD over the temperature range 290 ≤ *T* ≤ 320 K. Solid lines result from fits to eqn (12).

For each pH and temperature, the exchange rates *k*_D_ have been determined from three to seven ratios *I*_A_/*I*_B_ of the signal intensities corresponding to six to fourteen experiments performed with variable numbers of π-pulses 2 ≤ *n* ≤ 8 in the CPMG trains, and different intervals, *τ* = 2.6, 5.3, 10.6 and 21.2 ms, but with the same total relaxation time 2*τn*_CPMG_. A minimum of two ratios *I*_A_/*I*_B_ at different delays are required for an unambiguous determination of *k*_D_, since two rates can be compatible with a single *I*_A_/*I*_B_ ratio. Fig. 3 shows how this ambiguity is lifted by changing the inter-pulse delay 2*τ* in the CPMG pulse train. The pseudo first-order exchange rate constants were found to lie in a range 0 < *k*_D_ < 40 000 s^–1^, depending on pH and temperature (Table 1). At each temperature, the exchange rate *k*_D_ was found to be slowest for pD_min_ 4.8. When the exchange rate *k*_D_ is very low, one cannot neglect contributions due to the difference in relaxation rates of the in-phase ^15^N coherence and other rates in the relaxation matrix of eqn (6). From an earlier study of the exchange of indole protons,^7^ we know that the exchange rate *k*_H_ almost vanishes near pH_min_. On the other hand, as can be seen in Table 1, the exchange rates *k*_D_ do not vanish near pD_min_. Moreover, if one neglects relaxation of deuterium, some apparent exchange rates increase at lower temperatures, which is physically impossible. Hence, we incorporated a temperature-dependent quadrupolar relaxation rate *R*_Q_ in eqn (12) and subtracted it from the apparent exchange rates at all pD. The use of a single constant *R*_Q_ to describe the effects of deuterium relaxation is rather naive. In particular for weak *rf* fields or large deuterium offsets, this assumption may lead to errors. We can calculate the relaxation rates of operator products containing terms such as D_*z*_, (3D_*z*_^2^ – 2E), D_*x*_, D_*y*_, (D_*x*_^2^ – D_*y*_^2^), (D_*y*_D_*z*_ + D_*z*_D_*y*_) and (D_*x*_D_*z*_ + D_*z*_D_*x*_).^21^ However, we have verified that under the conditions for which the rates of Fig. 4 were obtained, *i.e.*, for strong *rf* fields and vanishing deuterium offsets, the exchange rates are barely affected if we assume that all deuterium terms have a common relaxation rate. The errors in the experimental ratios *I*_A_/*I*_B_ were determined from standard deviations. The error propagation was further simulated by the Monte Carlo technique. The errors in the exchange rates *k*_D_ were estimated from the curvature around the minima of *χ* ^2^ and found to lie in a range between 3 and 28%.

If the exchange rate constants *k*_D_ are plotted as a function of pD on a logarithmic scale, one obtains a V-shaped curve that is characteristic of acid catalysis by D^+^ ions and basic catalysis by OD^–^ ions, the latter being more efficient (Fig. 4). In the cationic, zwitterionic and anionic forms of tryptophan, the exchange rates result from sums of acidic and basic contributions. The overall exchange rate constant *k*_D_ can be written as:^2,22^

**Fig. 5:**
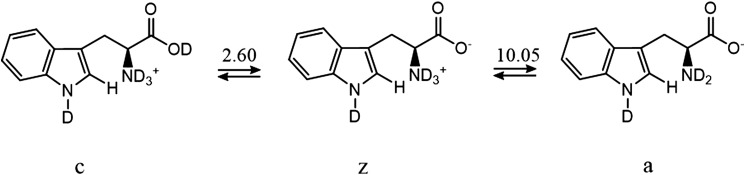
Tryptophan exists in three forms *c* (cationic), *z* (zwitterionic) and *a* (anionic), with mole fractions *f*_*c*_, *f*_*z*_ and *f*_*a*_ that depend on pD.

12 *k*_D_ = *k*cD*f*_c_[D^+^]_c_ + *k*zD*f*_z_[D^+^]_z_ + *k*zOD*f*_z_[OD^–^]_z_ + *k*aOD*f*_a_[OD^–^]_a_ + *R*_Q_

where the rate *R*_Q_ expresses contributions due to the quadrupolar deuterium relaxation to the decay of antiphase ^15^N coherences. The indices D and OD represent the contributions of acidic and basic mechanisms (see below) for the cationic, zwitterionic and anionic forms of tryptophan, abbreviated by *c*, *z*, and *a* in Fig. 5.

The mole fractions *f*_*c*_, *f*_*z*_ and *f*_*a*_ of the cationic, zwitterionic and anionic forms of tryptophan are:*f*_*c*_ = (1 + 10^pD–p*K*_a1_^ + 10^2pD–p*K*_a1_–p*K*_a2_^)^–1^ *f*_*z*_ = (1 + 10^–pD+p*K*_a1_^ + 10^pD–p*K*_a2_^)^–1^

13 *f*_*a*_ = (1 + 10^–pD+p*K*_a2_^ + 10^–2pD+p*K*_a1_+p*K*_a2_^)^–1^

Where [D^+^] = 10^–pD^, [OD^–^] = *K*_W_10^pD^. The auto-ionization constant *K*_W_ of D_2_O depends on the temperature.^23^ In H_2_O at 25 °C, p*K*_a1_ = 2.46 for the protonation of the carboxyl group, while p*K*_a2_ = 9.41 corresponds to the protonation of the amine group. In D_2_O at 25 °C, we have determined that p*K*_a1_ = 2.60 and p*K*_a2_ = 10.05.^24^ The variation of p*K*_a_ with temperature^23^ has been taken into account. Fig. 4 and Table 2 show the results of the fitting of the exchange rate constants *k*_D_ to eqn (12), which allows one to obtain the catalytic rate constants for the contributions of acid and basic mechanisms for each of the three forms *c*, *z*, and *a*. The basic contribution of the cationic form and the acidic contribution of the anionic form are masked by other terms and can be neglected.

**Table 2:** Exchange rate constants *k*_D_ and *k*_H_ [s^–1^] derived by fitting to eqn (12)

	290 K	300 K	310 K	320 K		300 K	310 K	320 K
*R*_Q_	36.2 ± 5.6	26.51 ± 3.7	19.4 ± 9.7	16.6 ± 3.3	*R*_Q_	0.37 ± 0.03	^*a*^	^*a*^
log(*k* *c*D)	2.91 ± 0.64	3.31 ± 0.27	3.49 ± 0.32	3.01 ± 1.20	log(*k* *c*H)	2.91 ± 0.04	^*a*^	^*a*^
log(*k* *z*D)	3.80 ± 0.95	3.74 ± 0.64	3.70 ± 1.22	4.14 ± 0.24	log(*k* *z*H)	3.31 ± 0.05	^*a*^	^*a*^
log(*k* *z*OD)	7.89 ± 0.07	8.10 ± 0.06	8.28 ± 0.03	8.41 ± 0.08	log(*k* *z*OH)	8.13 ± 0.02	8.29 ± 0.29	8.47 ± 0.41
log(*k* *a*OD)	6.64 ± 0.20	6.69 ± 0.30	6.87 ± 0.12	7.19 ± 0.19	log(*k* *a*OH)	7.53 ± 0.05	7.72 ± 0.27	7.97 ± 0.37

^*a*^Proton exchange rates were not measured at these temperatures.^7^

The activation energy *E*_a_ of the transition state provides a measure of the strength of N–D or N–H bonds.^25^ The activation energy *E*_a_ is defined by the Arrhenius equation

14 *k* = *A*e^–*E*_a_/*RT*^

where *A* is an empirical pre-exponential “frequency factor”, *R* the universal gas constant, *T* the temperature and *k* the exchange rate. The dependence of *E*_a_ on pH or pD for H–H and D–D exchange processes and the activation energies and pre-exponential frequency factors are shown in Table 3 for protons and in Table 4 for deuterium.

**Table 3:** Activation energies *E*_a_ and pre-exponential frequency factors *A* for the indole proton H^N^ in tryptophan

pH	*E*_a_ [kJ mol^–1^]	ln(*A*)
6.3	88 ± 2	37 ± 1
7.41	84 ± 2	37 ± 1
8.31	83 ± 4	39 ± 1
9.08	82 ± 6	40 ± 2
10.01	86 ± 14	43 ± 5
10.6	94 ± 12	47 ± 4

**Table 4:** Activation energies *E*_a_ and pre-exponential frequency factors *A* for the indole deuterium D^N^ in tryptophan. The activation energies and the pre-exponential factors are strongly correlated

pD	*E*_a_ [kJ mol^–1^]	ln(*A*)
1.0	38 ± 17	20 ± 7
1.5	40 ± 12	20 ± 5
2.0	35 ± 11	17 ± 4
7.0	87 ± 3	35 ± 1
8.0	86 ± 8	37 ± 3
9.0	82 ± 9	38 ± 3
10.0	73 ± 14	36 ± 6
11.0	72 ± 7	36 ± 3
12.0	87 ± 23	44 ± 9

One can speak of a kinetic isotope effect when the exchange rate is affected by isotopic substitution.^26^ In the present case, we compare the exchange rates of indole protons in tryptophan with H_2_O on the one hand, and analogous exchange rates of indole deuterons with D_2_O on the other. The kinetic isotope effect is defined as the ratio of the rate constants *k*_H_/*k*_D_. The change in exchange rates results from differences in the vibrational frequencies of the N–H or N–D bonds formed between ^15^N and ^1^H or ^2^D.^27–29^ Deuterium will lead to a lower vibrational frequency because of its heavier mass (lower zero-point energy). If the zero-point energy is lower, more energy is needed to break an N–D bond than to break an N–H bond, so that the rate of the exchange will be slower. Moreover, one expects *E*_a_ to be larger for deuterium. The results in Tables 3 and 4 do not support this expectation, but if one assumes the same pre-exponential frequency factor for H and D, *E*_a_ is indeed larger for the heavier isotope.

**Fig. 6:**
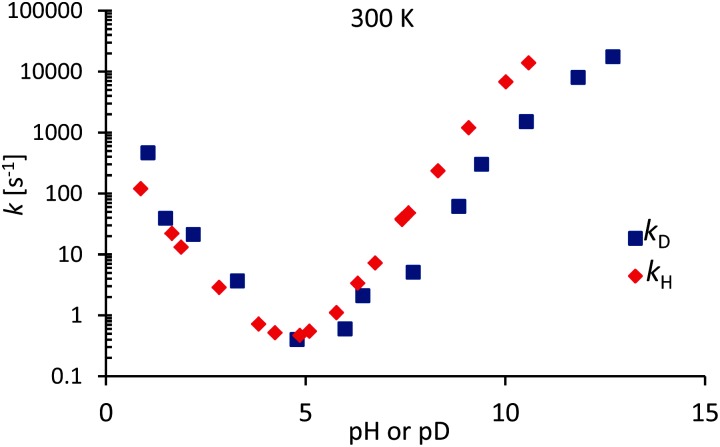
Deuterium and proton exchange rates *k*_D_ (blue) and *k*_H_ (red) as a function of pH or pD at 300 K. The pD scale has been corrected according to eqn (11) to take into account the use in D_2_O of a glass electrode designed for H_2_O.

Fig. 6 shows exchange rates *k*_D_ and *k*_H_ at 300 K. For acid catalyzed exchange, *k*_D_/*k*_H_ > 2.5 because D_3_O^+^ is a stronger acid than H_3_O^+^. For base catalyzed exchange, *k*_D_/*k*_H_ < 1. However, to compare the difference between catalysis by OH^–^ and OD^–^, we need to take into account the difference of the ionization constants: p*K*_W_(D_2_O) = 14.95 and p*K*_W_(H_2_O) = 13.99 at 25 °C.

**Fig. 7:**
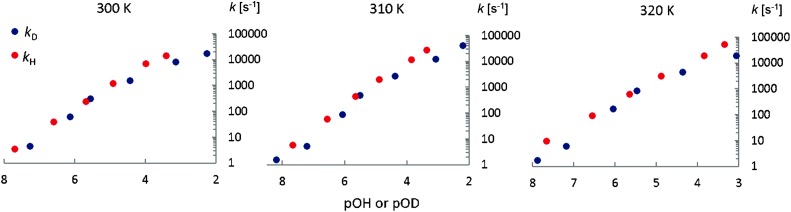
Base catalyzed exchange rates *k*_D_ and *k*_H_ as a function of pOH or pOD at different temperatures.

Fig. 7 shows the base-catalyzed exchange rate constants *k*_D_ and *k*_H_ as a function of pOH or pOD. The exchange rates *k*_D_ are slightly lower than *k*_H_, giving the approximate kinetic isotope effects: *k*_H_/*k*_D_ = 2.2 ± 0.3, 2.3 ± 0.3 and 2.1 ± 0.3 at 300 K, 310 K and 320 K respectively (Fig. 7.) These values result from averages of the exchange rate constants for the zwitterionic and anionic forms (Table 5).

**Table 5:** Kinetic isotope effects (KIE) *k*_H_/*k*_D_ for the exchange rate constants of each of the three forms of tryptophan in solution: *c* (cationic), *z* (zwitterionic), and *a* (anionic)

	300 K	310 K	320 K
*k* *c* H /*k* *c*D	0.40 ± 0.04	^*a*^	^*a*^
*k* *z* H /*k* *z*D	0.37 ± 0.09	^*a*^	^*a*^
*k* *z* OH /*k* *z*OD	1.1 ± 0.1	1.0 ± 0.3	1.1 ± 0.5
*k* *a* OH /*k* *a*OD	7 ± 2	7 ± 2	6 ± 3

^*a*^Proton exchange rates were not measured at these temperatures.^7^

In Table 5 the KIE is defined as *k* *i*H/*k* *i*D for acid catalysis or as *k* *i*OH/*k* *i*OD for base catalysis, where *i* = *c*, *z*, and *a* stand for the cationic, zwitterionic, and anionic forms of tryptophan in solution, with the heaviest isotope always in the denominator. If tunneling can be neglected, the KIE depends on the nature of the transition state. The maximum isotope effect for N–H bonds is *k*_H_/*k*_D_ ≈ 9, assuming that the bond is completely broken in the transition state (TS). The KIE can be reduced if the bonds are not completely broken in the TS. The KIE can be close to 1 if the TS is very similar to the reactant (N–D bond nearly unaffected) or very similar to the product (N–D bond almost completely broken).

The experimental ratio *k* *a*OH/*k* *a*OD is near its maximum when pH > p*K*_a2_, which suggests that the N–D bond is broken in the rate-limiting step and that the deuteron is half-way between the donor and the acceptor. However the ratio *k* *z*OH/*k* *z*OD ≈ 1 suggests that the N–D bond is either only slightly or almost completely broken in the TS. The protonation of the amine withdraws electron density and increases the acidity of the H^N^ group which favors the formation of the anionic form. This explains why *k* *z*OH/*k* *a*OH > 1 and *k* *z*OD/*k* *a*OD > 1. For the acid-catalyzed exchange constants, we observe an inverse kinetic isotope effect. This can happen when the degree of hybridization of the reactant is lower than that of the reaction center in the TS during the rate-limiting step.

**Fig. 8:**
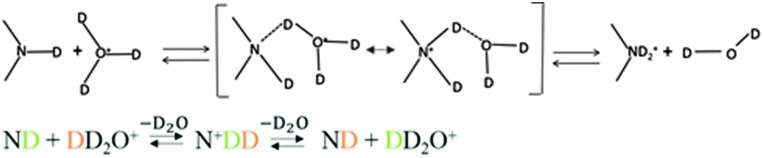
Acid-catalyzed mechanism of exchange. The transition state is shown in brackets.

**Fig. 9:**
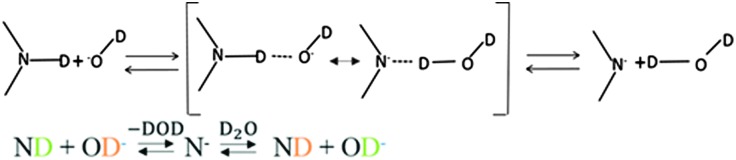
Base-catalyzed mechanism of exchange. The transition state is shown in brackets.

The mechanisms for proton or deuteron exchange have been thoroughly reviewed.^30–32^ Englander^30^ and his collaborators pointed out that the rate of the exchange of protons attached to nitrogen depends on the ability to form hydrogen-bonded complexes in the transition state involving the donor (tryptophan) and the acceptor (D_2_O or OD^–^). This occurs in three steps: (i) encounter of the donor and the acceptor, (ii) formation of the transition state involving the donor and acceptor, and (iii) cleavage of the N–D bond. The mechanism of acid-catalyzed exchange consists of the addition onto the nitrogen of a D^+^ ion from the solvent, followed by removal of D^+^ by D_2_O (Fig. 8). The mechanism of the base-catalyzed reaction involves removing the indole deuterium to create the conjugate base, which then abstracts a D^+^ from D_2_O to regenerate the indole (Fig. 9).

Altogether we can say that the rate-limiting step in the base-catalyzed mechanism is the removal of the proton or deuteron from the nitrogen. On the other hand, for the acid-catalyzed mechanism, is the donation of a proton or deuteron by H_3_O^+^ respectively D_3_O^+^. Finally, the curves of log *k*_D_ *vs.* pD and of log *k*_H_ *vs.* pH show a combination of specific base catalysis at high pH, and a specific acid catalysis at low pH, which becomes more important at higher temperatures.

## Conclusions

We have adapted our method that was originally designed for measuring fast H–H exchange rates *k*_H_ to the study of D–D exchange rates *k*. In tryptophan in aqueous solution over a range of pH, respectively pD, the kinetic isotope effect, defined as the ratio *k*_H_/*k*_D_ between the H–H and D–D exchange rates, was determined at several temperatures. The dependence of the activation energies on pH provides new insight into the mechanisms of the exchange processes. The results agree with the mechanisms discussed by Englander *et al.* ^30^

## Abbreviations

CPMG: Carr Purcell Meiboom Gill
KIE: Kinetic isotope effect
TS: transition state
